# Supplementary material for: The interactome of intact mitochondria by cross-linking mass spectrometry provides evidence for coexisting respiratory supercomplexes
Source: Mol Cell Proteomics. 2017 Dec 8;17(2):216–32. doi: 10.1074/mcp.RA117.000470 (PMC5795388; doi:10.1074/mcp.RA117.000470)
Supplement: Supplemental Data [file supp_RA117.000470_133922_0_supp_23978_fzffwf.zip › spectra_annotation/mito_DR_spectra_annotation/433-1-5-1-11-1.pdf]

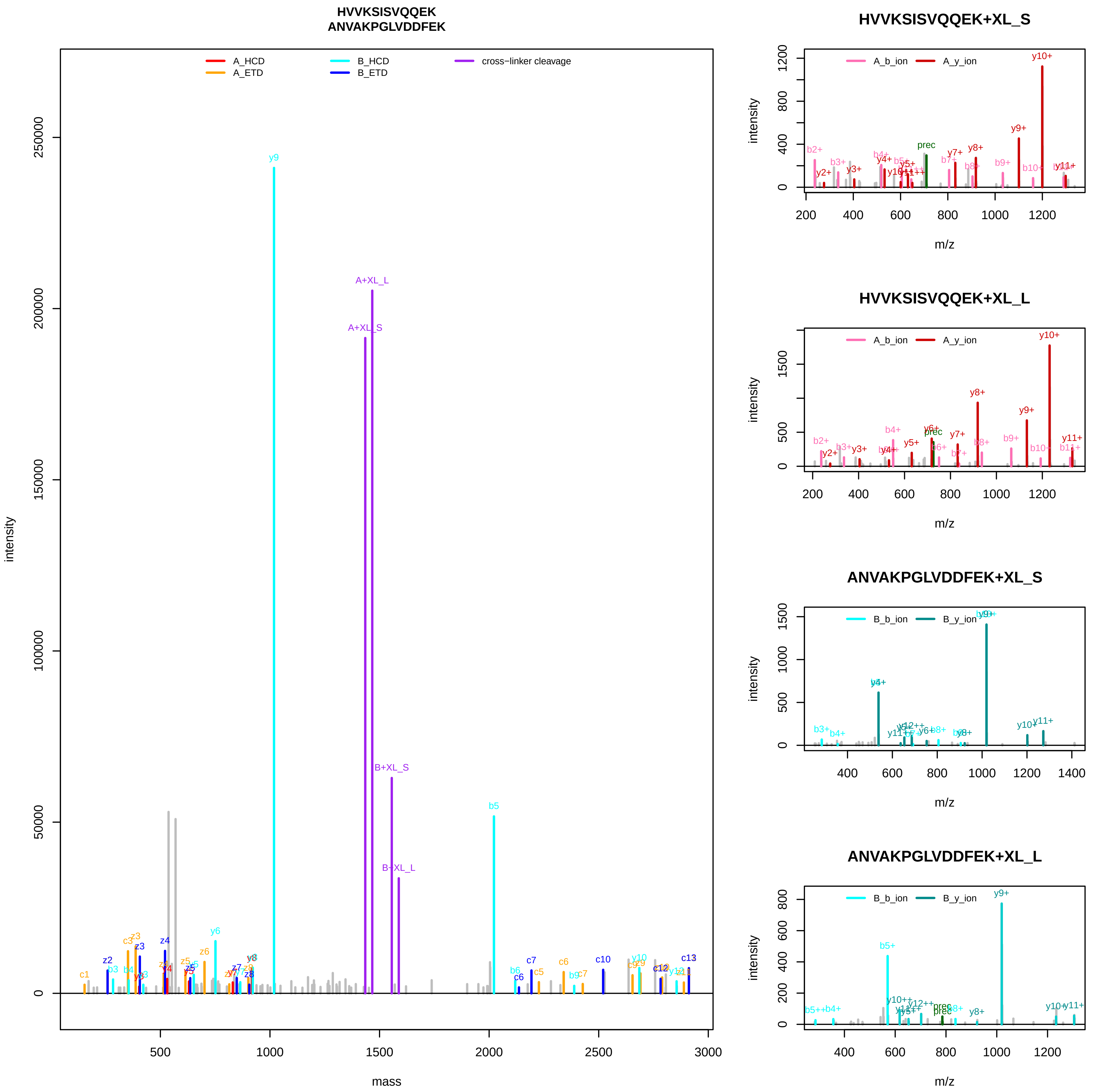
HVVKSISVQQEK
ANVAKPGLVDDFEK
A_HCD
A_ETD
B_HCD
B_ETD
cross−linker cleavage
intensity
mass
y9
A+XL_L
A+XL_S
B+XL_S
B-XL_L
b5
y6
z4
z3
z2
c3
c1
c7
c10
c13
c6
c5
c12
y10
HVVKSISVQQEK+XL_S
HVVKSISVQQEK+XL_L
ANVAKPGLVDDFEK+XL_S
ANVAKPGLVDDFEK+XL_L
A_b_ion
A_y_ion
B_b_ion
B_y_ion
prec
y10+
y9+
y8+
y11+
b5+
y4+
m/z
